# Mental health stigma among university health care students in Nigeria: a cross-sectional observational study

**DOI:** 10.11604/pamj.2020.37.5.24898

**Published:** 2020-09-02

**Authors:** Aderonke Bamgbose Pederson, Inger Burnett-Zeigler, Joyce Konadu Fokuo, Katherine Leah Wisner, Katelyn Zumpf, Yewande Oshodi

**Affiliations:** 1 Northwestern University, Chicago, Illinois, United State of America,; 2 University of California in San Francisco, San Francisco, California, United State of America,; 3 Lagos University Teaching Hospital, University of Lagos, Lagos, Nigeria

**Keywords:** Stigma, mental health, African, university students, religion

## Abstract

**Introduction:**

stigma is a key barrier to access and utilization of mental health services, particularly in low- and middle- income countries. The authors explore the specific content of mental health stigma among Nigerian university health care students at a national teaching hospital. These students are key stakeholders and represent a vital demographic to engage in stigma reduction initiatives. We evaluated the extent to which demographic characteristics, mood symptoms and utilization of resources are associated with stigma.

**Methods:**

the authors examined data obtained from surveys completed by university health care students (N = 82) at Lagos University teaching hospital. Surveys assessed demographic background, mood symptoms and use of mental health services. Simple linear regression was used to model the unadjusted association between each component variable and overall stigma score. All analyses were conducted using R (version 3.5.3, 2019, The R Foundation) and assumed a two-sided, 5% level of significance.

**Results:**

being a member of the minority ethnic group within our study population was associated with increased stigma. Individuals having greater need for mental health services (due to mood symptoms) were associated with increased stigma. Willingness to use medical services and community support from family and friends was associated with lower stigma. Religious themes were prominent among the majority of respondents.

**Conclusion:**

consideration of the content details of stigma among university health care students in Nigeria is essential to inform interventions and strategies to reduce stigma within this subgroup. Those students who have symptoms of depression or anxiety may have lower utilization of mental health services.

## Introduction

Stigma is a key barrier to utilization of mental health services [1]. Stigma is defined as the co-occurrence of labeling, stereotyping, separation, status loss and discrimination in the context of a power differential [2]. Mental health stigma refers to having negative thoughts, beliefs and discriminatory behaviors towards individuals with mental illness or those receiving mental health services [3]. Public stigma is generalized to community members´ conceptualization of society´s values towards those with mental illness, and self-stigma is related to the unique thoughts, attitudes and beliefs an individual has about mental illness [3, 4]. Internalized stigma is defined as stigma an individual may have towards his/her own mental illness [5]. Understanding and assessing the content of stigma is essential to building effective stigma reducing interventions and promoting help seeking behaviours [1, 6].

Approximately 450 million people worldwide suffer from neuropsychiatric conditions [7, 8]. In Africa, neuropsychiatric conditions account for 17.6% of all years of life lived with a disability, which presents a critical public health burden [7]. In Nigeria, the mental health system has limited skilled personnel to treat the population, with one psychiatrist for every one million people [7] and 150 psychiatrists for a population of 150 million people [9]. In Nigeria, the presence of stigmatizing views about mental health results in lower utilization of mental health services and poorer health outcomes [9, 10]. In the first large scale epidemiological survey of mental disorders in Nigeria, only 1% of those with DSM IV disorders received specialist mental health services. Even with inclusion of general medical services, only 9% of individuals with any 12-month DSM IV disorder received treatment [10, 11]. The gap between the prevalence of mental illness and provision of care is expansive and elimination of stigma in critical to narrow this gap.

To understand the challenge of eliminating stigma, understanding the nature and characteristics of stigma is essential [12, 13]. Limited studies that delineate the content and scope of mental health stigma beyond the general existence of stigma have been published. Even fewer studies of acculturation factors and cultural variations that influence mental health stigma are available [6, 14]. Some medical students and health professionals in Nigeria hold stigmatizing attitudes and beliefs towards persons with mental illness [15, 16]. The presence of religious and cultural frameworks specific to Nigerians shape the conceptualization of mental illness [11, 17, 18]. Nigeria is made up of 374 ethnic groups across 36 states, three ethnic groups form the majority of the country´s population; Hausa, Yoruba and Igbo ethnic groups represent 70% of the country´s population [19]. All three groups are influenced by both western religion and traditional belief systems. Across all groups, many believe mental illness is caused by weakness, spiritual punishment or moral failings [17]. However, to our knowledge, the content of mental illness stigma and its relationship to demographic factors, mood symptoms and preferred utilization of resources for mental health among university health care students have not been published.

The purpose of this exploratory study was to assess stigma among university health care students at a major national university in Nigeria, located in a predominantly Yoruba region. We aimed to investigate the characteristics of mental health stigma among university health care students and determine its relationship with demographic factors, mood symptoms and attitudes towards the utilization of resources. The goal was to understand the components of stigma in this population, which will inform the development of appropriate and culturally sensitive interventions that are targeted and effective among Nigerian university medical students. Among younger people, education can be the most effective tool to reduce stigma [20]. Expanding curriculum development around mental health and stigma is a feasible and effective next step among Nigerian medical students.

## Methods

### Study design and procedure

**Recruitment**: all study activities took place at Lagos University teaching hospital, which is part of the University of Lagos in Nigeria, one of the major universities in West Africa. This study was conducted in March 2019 as part of a university wide seminar on mental health awareness. Flyers and bulletins were placed in public announcement spaces. All 116 registered students participating in the seminar were invited to complete the survey on a voluntary basis; 82 (71%) completed the surveys after informed consent was obtained. Time was allotted as part of the seminar to complete the survey.

**Ethics review:** the study was approved by the internal review board at Northwestern University and the Research and Ethics committee at Lagos University Teaching Hospital. **Inclusion criteria**: all participants had an affiliation to the university community. Persons who were university students, between ages 18-65 years old and English speaking were eligible to participate (N = 82). The study included both undergraduate and graduate students within the medical school system including medical students, nurses, pharmacists and other health care students. In Nigeria, students begin university on average at age 16 years; students under 18 years were excluded to ensure they met criteria for consent based on both ethics review boards at the two parent institutions.

**Measures:** we administered the following measures in English: 1) socio-demographic questionnaire assessed age, gender, race/ethnicity, education, employment, income, insurance and marital status. 2) a self-report 37-question stigma survey developed by the first author (AOBP), the stigma and culture survey (SCS). The questions were based on existing stigma scales, the depression self-stigma scale (DSSS) and the internalized stigma of mental illness scale (ISMI) [21, 22]. This scale extended existing measures by assessing views on prayer, spirituality, cultural and religious factors specific to a Nigerian community. We sought to capture stigmatizing views that were not based on Western conceptualization of mental illness. In addition, although both the DSSS and ISMI assume the respondents have a mental illness, our scale was administered without this assumption. Public stigma was measured using questions about general views of mental illness. Self-stigma was measured using questions about unique thoughts, attitudes and beliefs an individual has about mental illness. The survey also included questions about depression, stress or anxiety, mechanisms to manage mental health and preferred use of resources. A Likert scale was used with responses of never, rarely, sometimes, very often and always. The full questionnaire is available from the corresponding author upon request.

**Statistical analysis:** the survey did not require participants to complete all questions. To address missingness, stigma scores were defined as the average rather than summation of subjects´ responses. Unless specified as a reverse-coded item, all responses were coded as 1 for “never”, 2 for “rarely”, 3 for “sometimes”, 4 for “very often”, and 5 for “always”. We defined self-stigma as the average response for questions 6, 8, 9, 14, 17, 18, 19, 20, 21, 22, 24, 25, 26, 28, 33, 34, 35, and 36. Questions 14, 22, 24, 25, 26, and 28 were reverse coded. Public stigma was defined as the mean response for questions 7, 16, 5, and 11, with the latter two questions reverse coded. Overall stigma score was calculated as the average of self- and public-stigma related questions. Scores ranged from 1 to 5 with higher stigma scores representing greater stigmatizing views. We separated the components of stigma into self-stigma and public stigma. To reduce the effect of social desirability we provided participants with privacy to complete their surveys and anonymity of their responses. To describe the sample, we recorded frequency and percent of all categorical variables and both mean ± standard deviation, median, and range for continuous variables. Simple linear regression was used to model the unadjusted association between each demographic variable and overall stigma score. Type III ANOVA was performed to evaluate the overall association between multinomial categorical variables and stigma. We repeated this method for secondary outcomes: self and public stigma. We conducted all analyses using R (version 3.5.3, 2019, The R Foundation) and assumed a two-sided, 5% level of significance. Since the study was exploratory, we did not adjust for multiple hypothesis testing [23].

## Results

Eighty-two participants (N = 82; 71%) completed our survey questionnaires. The characteristics of these participants are included in Table 1. The distribution among respondents of use of mental health resources available in the community is included in Table 2. The mean age of sample was 27.6 years and majority identified as single (70.7%), female (75.6%), Yoruba ethnic group (72%). Among the students, a majority identified as having some university level education or higher (81.7%). Socioeconomic status (SES) was defined based on monthly income, employment status and education level, grouped as high (25.6%), middle (30.5%) or low (14.6%). We assigned SES based on components of education, income and employment. Having monthly income > N10, 000 naira, having employment and having some university or higher education was assigned one point each. We defined high SES as having all three components, middle SES as having two of three components and low SES as having none or one component. We did not assign those with their one point being high monthly income to low SES.

**Table 1:** demographic characteristics of university health care students at Lagos University Teaching Hospital in Nigeria (N = 82)

Variables	Overall (n=82)
**Age**	
Mean (SD)	27.6 (9.39)
Median [Min, Max]	23.0 [18.0, 57.0]
Non-responders	2 (2.4%)
**What is your marital status?**	
married	24 (29.3%)
single	58 (70.7%)
**What is your gender? (other was an option)**	
Female	62 (75.6%)
Male	15 (18.3%)
Non-responders	5 (6.1%)
**Ethnic background**	
Igbo	17 (20.7%)
Other	4 (4.9%)
Yoruba	59 (72.0%)
Non-responders	2 (2.4%)
**Level of Education**	
WAEC or Less	11 (13.4%)
Some University	37 (45.1%)
Bachelor’s degree	20 (24.4%)
Some graduate School or More	10 (12.2%)
Non-responders	4 (4.9%)
**Employment Status**	
Not Employed	56 (68.3%)
Employed	26 (31.7%)
**Socioeconomic Status**	
Low	12 (14.6%)
Middle	25 (30.5%)
High	21 (25.6%)
Non-responders	24 (29.3%)

**Table 2:** distribution in use of mental health resources among health care students at Lagos University Teaching Hospital in Nigeria (N = 82)

Willingness to use resources to manage mental health	Overall (n=82)
**Religious Resources**	
Mean (SD)	3.42 (0.670)
Median [Min, Max]	3.33 [1.67, 5.00]
**Community, Family, Friend Resources**	
Mean (SD)	3.06 (0.697)
Median [Min, Max]	3.00 [1.00, 5.00]
**Biological, Formal Medical Resources**	
Mean (SD)	3.49 (0.788)
Median [Min, Max]	3.33 [1.33, 5.00]

**The association of demographic characteristics and stigma:** we found an association between stigma scores and ethnicity (Table 3). Yoruba participants had an overall, self and public stigma score that was lower, hence lower stigmatizing views on average compared to Igbo participants [-0.24, 95% CI (-0.45, -0.04), p = 0.02; -0.22, 95% CI (-0.44,-0.01), p = 0.042; -0.33, 95% CI (-0.65, -0.01), p = 0.043]. We did not find a significant correlation between age, gender, marital status and socio-economic status with stigma scores.

**Table 3:** simple linear regression results: evaluating the association between participant characteristics and mental health stigma (n = 82)

Variables	Overall Stigma Estimate (95% CI); p-value	Self-Stigma Estimate (95% CI); p-value	Public Stigma Estimate (95% CI); p-value
**Age**	0 ( -0.01 , 0.01 ); 0.974	0 ( -0.01 , 0.01 ); 0.851	0 ( -0.01 , 0.02 ); 0.63
**Marital Status** single vs married	0.12 ( -0.06 , 0.3 ); 0.198	0.13 ( -0.07 , 0.32 ); 0.194	0.09 ( -0.2 , 0.38 ); 0.526
**Gender** Male vs Female	0.19 ( -0.03 , 0.41 ); 0.089	0.18 ( -0.05 , 0.4 ); 0.131	0.25 ( -0.1 , 0.59 ); 0.156
**Ethnicity** Yoruba vs Igbo	-0.24 ( -0.45 , -0.04 ); 0.0201	-0.22 ( -0.44 , -0.01 ); 0.0424	-0.33 ( -0.65 , -0.01 ); 0.0432
**Socioeconomic Status** High vs Low	-0.18 ( -0.45 , 0.09 ); 0.194	-0.23 ( -0.5 , 0.05 ); 0.110	0.05 ( -0.41 , 0.5 ); 0.834
**Religious Resources**	0.09 ( -0.03 , 0.22 ); 0.144	0.1 ( -0.03 , 0.23 ); 0.117	0.04 ( -0.16 , 0.24 ); 0.701
**Community, Family, Friend Resources**	-0.07 ( -0.19 , 0.05 ); 0.28	0 ( -0.13 , 0.13 ); 0.990	-0.36 ( -0.53 , -0.19 ); 0
**Biological, Formal, Medical Resources**	-0.3 ( -0.39 , -0.22 ); 0	-0.33 ( -0.42 , -0.25 ); 0	-0.17 ( -0.34 , -0.01 ); 0.042
**Seek Help if Someone Has Suicidal Thoughts Very Often**-Always vs Never-Sometimes	-0.35 ( -0.58 , -0.11 ); 0.004	-0.45 ( -0.69 , -0.21 ); 0	0.08 ( -0.31 , 0.47 ); 0.674
**Depression Very Often**-Always vs Never-Rarely	0.47 ( 0.16 , 0.78 ); 0.003	0.47 ( 0.15 , 0.8 ); 0.005	0.44 ( -0.07 , 0.95 ); 0.089
**Q37 Government Interventions** Checked vs Not	-0.29 ( -0.49 , -0.09 ); 0.006	-0.3 ( -0.51 , -0.09 ); 0.005	-0.21 ( -0.54 , 0.12 ); 0.208

**The association between mood symptoms and stigma**: compared to participants who never or rarely felt sad, down, depressed, stressed, anxious, or nervous, those who frequently had these symptoms had a greater overall average stigma score or greater stigmatizing views [0.47, 95% CI: (0.16, 0.78), p = 0.004]. Compared to participants who never or rarely feel sad, down, depressed, stressed, anxious, or nervous, those who frequently had these symptoms also had a higher average self-stigma score [0.47, 95% CI: (0.15, 0.8), p = 0.005].

**Relationship between preferred use of resources and stigmaUse of resources and stigma outcomes:** we categorized the preferred use of resources for mental health needs into 3 components: medical (use of medication or hospital resources), religious (spiritual counseling) and community (seeking friend/family support). As use of biological or medical resources increased, overall stigmatizing views decreased [-0.3, 95% CI: (-0.39,-0.22), p < O.0001]. Similarly, using biological or medical resources was associated with lower self-stigma scores [-0.33, 95% CI: (-0.42,-0.25), 0001'> p<.0001] and lower public stigma scores [-0.17, 95% CI: (-0.34, -0.01), p= 0.042]. As use of community resources increased, public stigma decreased [-0.36, 95% CI: (-0.53, -0.19), p < 0.0001]. We did not find a statistically significant association between use of religious resources and stigma.

**Use of resources in case of emergencies and stigma outcomes:** we also assessed the likelihood of use of necessary resources (such as calling emergency services) in cases of psychiatric emergencies such as active suicidality and stigma. Compared to participants who would never or only sometimes seek immediate medical care for someone with suicidal thoughts, those who would often seek immediate care had a lower overall stigma score and hence lower stigmatizing views [-0.35, 95% CI: (-0.58, -0.11), p = 0.004]. The association between self-stigma and care seeking behavior for suicidality was similar [-0.45, 95% CI: (-0.69,-0.21), p < 0.0001].

**Mechanisms to reduce stigma:** we assessed preferred mechanisms for reduction of stigma and its association with overall stigma scores. Those who endorsed government interventions as a method to reduce negative feelings or shame about mental illness had lower average overall stigma scores and lower stigmatizing views [-0.29, 95% CI: (-0.49, -0.09), p = 0.006] than those who did not. This was similar with self-stigma [-0.3, 95% CI: (-0.51, -0.09), p = 0.005]. Stigma scores varied in those who believe more information at schools or universities, more information at work, public education and campaigns, family education, community education, or mental fairs could reduce mental health stigma in their communities.

**Figure 1:**
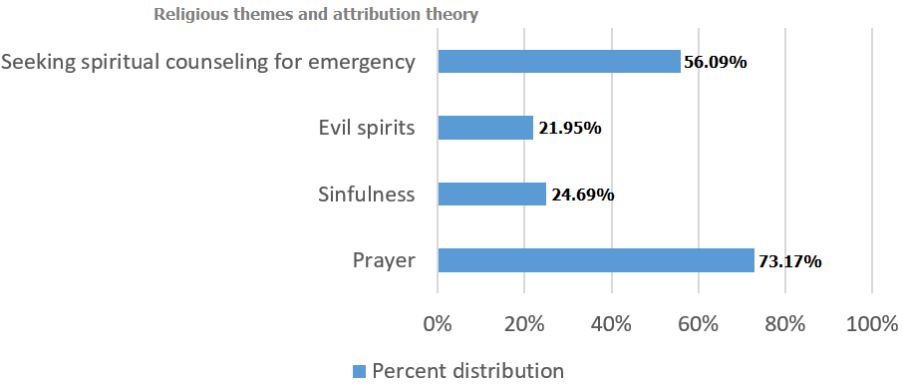
distribution of religious themes among health care students at Lagos University Teaching Hospital in Nigeria (N = 82)

**Religious themes and spiritual counseling within mental illness among students**: among respondents, 92.68% stated they would pray sometimes if they were feeling depressed or anxious and 73.17% stated they would pray most of the time (Figure 1). Nearly a quarter (24.69%) of respondents say mental illness is sometimes or often caused by sin, and 21.95% believe mental illness is sometimes caused by evil spirits. More than half (56.09%) of respondents would seek spiritual counseling for active suicidal ideation. When asked about seeking immediate medical care for active SI, 13.42% selected that they rarely would seek immediate medical care. Greater than one-third of the respondents would not always seek immediate medical care for active SI, considered a psychiatric emergency.

## Discussion

We found that participants who identified as Yoruba had lower stigma scores in all domains (overall, self and public). One possible reason for this is that 70% of our pool of participants were part of the majority ethnicity (Yoruba), as Lagos State is an ethnically Yoruba region. This indicates a possible relationship between lower stigma scores and being part of a majority ethnic group compared to being part of the minority group. The results may differ with more variation in ethnicity across the sample. Minorities are more likely to experience discrimination [6] which has implications for mental health service provision and how it is disseminated. Similarly, in western settings, minorities (Blacks and Hispanics) have worse mental health outcomes than the white majority [24, 25].

Respondents who reported symptoms such as frequently feeling down, anxious or depressed had higher overall scores and self-stigma scores. The presence of stigma has been associated with greater barriers to help seeking behaviors. Our analysis suggests that those who have mood symptoms have higher stigma scores and hence greater stigmatizing views. Individuals with the greater need for mental health services are less likely to access these services due to the stigma associated with mood symptoms [1]. Respondents who preferred using biological or medical resources had lower stigma scores. This was consistent with their willingness to seek immediate medical care for active suicidality. As use of emergent psychiatric services increased, stigma scores decreased. Although not statistically significant, those who preferred religious resources had higher stigma scores than those who preferred biological or medical resources. The content of mental health stigma among Nigerians includes spiritual beliefs, possession by demons and a religious framework which impacts the use of mental health services in Nigeria [17, 26]. The use of resources - biological/medical, community/family, religious - warrants further investigation in its relationship to stigma.

The emphasis of spirituality in understanding and conceptualizing mental illness is a characteristic of many Nigerians; therefore, addressing stigma may be akin to challenging an individual´s personhood [6]. Those who believe prayer is important to addressing depressed or anxious feelings were the majority of respondents. Almost a quarter of respondents believe mental illness is a sin and one in five believe mental illness is sometimes caused by evil spirits. More than half of respondents would seek spiritual counseling for active suicidal ideation, a psychiatric emergency. These belief systems likely contribute to the mortality and morbidity related to mental illness in low income countries [7, 8], and warrant further investigation because religion plays an essential role in health [27]. The intersection of religion and care seeking behaviors among those with mental illness in low-income countries would contribute to addressing barriers to utilization of mental health services. We assessed preferred mechanisms for reduction of stigma and their association with overall stigma scores. Compared to those who do not believe government interventions would be effective, those who believe government interventions would be effective as a preferred method to reduce negative feelings or shame about mental illness had lower average stigma scores and thus lower stigmatizing views. In Nigeria, the policies around mental health are limited [9]. Government interventions rely on educational programs and mass media campaigns [28] to reduce the negative stereotypes portrayed in the news media [29, 30]. Authors of a 2014 meta-analysis reported that education is the most common type of stigma reducing intervention employed [5]. However, while education is the most effective for young people, among adults social contact has greater efficacy [5, 31, 32]. Further investigation is warranted to explore government based interventions among students in Nigeria, which has been successful with infectious disease intervention campaigns within school systems [33, 34]. Insufficient evidence exists for mass media interventions in middle and low income countries [28], which also warrants further consideration.

The willingness to use biological or medical resources correlates strongly with lower mental health stigma, which suggests that stigma reduction and willingness to use these resources will lead to increased utilization [1, 6]. A negative correlation was observed between use of family/friend resources to manage mental health and public stigma. This finding suggests that those who would disclose mental health information to family and friends have less public stigma [30]. We observed an association between use of medical resources and lower public stigma scores. Interventions to reduce public stigma may lead to increased use of medical resources. We assessed whether we could identify factors that suggest methods for stigma reducing interventions. We found age and education did not significantly predict ways to reduce negative mental illness stigma. This is relevant because educational interventions to reduce stigma are more effective in young people compared to adults [31]. Since this was an exploratory pilot study with a relatively small sample size, we were unable to adjust for potential confounding through covariate adjustment or propensity score weighting. We plan to use these preliminary findings to conduct a larger study in the future and consider mediating and/or moderating factors in the association between demographic factors, mood symptoms, use of resources and stigmatizing views. The study provides preliminary data that will contribute to future studies. We did not seek to measure stigma among those with known psychopathology, hence for our depression and anxiety screening question, we used a single question on a Likert scale to reduce responder burden. In a future study, we plan to conduct more in-depth mood assessments with a validated questionnaire such as the PHQ-9. Using a standardized and validated screen for depression would allow us to describe further the mood symptoms within the population. For definitions that may have been vague, we clarified meanings; for example, biological or medical resources, were described within the questionnaire as using resources such as seeking medical care, seeking care in a hospital setting, and use of medications. For this exploratory study, we sought to identify broad perspectives on use of mental health resources. In a future study, we plan to delineate different forms of treatment resources including alternative treatment modalities like psychotherapy, mindfulness or light therapy and the extent to which stigmatizing views influence use of specific treatment resources.

## Conclusion

In conclusion, we highlight several factors in this exploratory study that influenced the level of stigma expressed within this group of university health care students who are key stakeholders in advancing care of mental illness for Nigerians. Exploring stigma among future health care providers is essential. The findings from this study can shape future stigma reducing interventions and influence strategies to build stigma reducing interventions for use within medical schools locally in Nigeria and broadly in low- to middle- income countries with similar conceptualizations of mental illness. We hope this will improve mental health outcomes among Nigerians particularly where stigma is a key barrier to care.

### What is known about this topic

- Mental health stigma is a barrier to utilization of mental health services;
- Stigma is present among clinicians;
- There is limited data from low to middle income countries on mental health stigma.

### What this study adds

- Within this sample, ethnic minorities were at greater risk of higher stigmatizing views, there is limited data on ethnic minorities and mental health stigma among health care students within this cultural context;
- University health care students who may have low mood may be more likely to have stigmatizing views despite a greater need for mental health support services;
- Spirituality and religiosity is a key component of conceptualizing mental health and needs to be incorporated into interventions to reduce stigma among future health care providers.
